# APOE3 Christchurch influences amyloid induced tau seeding and spreading via effects on microglia

**DOI:** 10.1002/alz.086126

**Published:** 2025-01-03

**Authors:** Yun Chen, Sihui Song, Samira Parhizkar, Jennifer Lord, Yiyang Zhu, Michael R Strickland, Chanung Wang, Jiyu Park, G. Travis Tabor, Hong Jiang, Kevin Li, Albert A Davis, Carla M Yuede, Marco Colonna, Jason D Ulrich, David M. Holtzman

**Affiliations:** ^1^ Washington University in Saint Louis, Saint Louis, MO USA; ^2^ Washington University in St. Louis, St. Louis, MO USA; ^3^ Washington University School of Medicine, St. Louis, MO USA; ^4^ Washington University in St. Louis, SAINT LOUIS, MO USA; ^5^ Knight ADRC, Saint Louis, MO USA; ^6^ Hope Center for Neurological Disorders, Saint Louis, MO USA; ^7^ Hope Center for Neurological Disorders, Washington University, St. Louis, MO USA; ^8^ Washington University School of Medicine, Saint Louis, MO USA; ^9^ Washington University School of Medicine, St Louis, MO USA; ^10^ Department of Neurology, Washington University School of Medicine, St. Louis, MO USA; ^11^ Hope Center for Neurological Disorders, Washington University School of Medicine, St. Louis, MO USA; ^12^ Knight Alzheimer Disease Research Center, St. Louis, MO USA; ^13^ Washington University School of Medicine in St Louis, St. Louis, MO USA

## Abstract

**Background:**

A recent case report described an individual who was a homozygous carrier of the APOE3 Christchurch (APOE3ch) mutation and resistant to autosomal dominant Alzheimer’s Disease (AD) caused by a PSEN1‐E280A mutation. Whether APOE3ch contributed to the protective effect remains unclear.

**Method:**

We generated a humanized APOE3ch knock‐in mouse and crossed it to an amyloid‐β (Aβ) plaque‐depositing model. We injected AD‐tau brain extract to investigate tau seeding and spreading in the presence or absence of amyloid. Mouse samples were collected and used for multiple measurements including cholesterol test, WB, ELISA, IF, etc. We also designed and performed in vitro assays to evaluate the phagocytosis, degradation, competitive uptake, exocytosis, etc.

**Result:**

APOE3ch expression resulted in peripheral dyslipidemia and a marked reduction in plaque‐associated tau pathology. Additionally, we observed decreased amyloid response and enhanced microglial response around plaques. We also demonstrate increased myeloid cell phagocytosis and degradation of tau aggregates linked to weaker APOE3ch binding to heparin sulfate proteoglycans in vitro.

**Conclusion:**

APOE3ch influences the microglial response to Aβ plaques, which suppresses Aβ‐induced tau seeding and spreading.

